# *SARS-CoV-2* infection increases airway bleeding risk in patients after tracheostomies

**DOI:** 10.1186/s12985-024-02320-2

**Published:** 2024-03-07

**Authors:** Shupin Tang, Gongbiao Lin, Xiaobo Wu, Zhihong Chen

**Affiliations:** 1 Department of Otorhinolaryngology Head and Neck Surgery, The First Affiliated Hospital, Fujian Medical University, Fuzhou, 350005 China; 2 Department of Otorhinolaryngology Head and Neck Surgery, National Regional Medical Center, Binhai Campus of the First Affiliated Hospital, Fujian Medical University, Fuzhou, 350212 China; 3 Fujian Institute of Otorhinolaryngology, The First Affiliated Hospital, Fujian Medical University, Fuzhou, 350005 China

**Keywords:** *SARS-CoV-2*, Tracheostomy, Airway bleeding

## Abstract

**Background:**

Airway bleeding events are a rare incident in *SARS-CoV-2*-infected patients after tracheostomies. We aimed to explore the correlation between airway bleeding and *SARS-CoV-2* infection and evaluate the consistency of *SARS-CoV-2* RNA test results in the upper and lower airway samples from patients after tracheostomies.

**Methods:**

Forty-four patients after temporary or permanent tracheostomy were divided into a positive group (29 patients) and a negative group (15 patients) based on the *SARS-CoV-2* RNA test results of their oropharyngeal swabs. The oropharyngeal and tracheal swabs of the positive group were re-collected for *SARS-CoV-2* RNA detection. Demographic and clinical characteristics and airway bleeding events were recorded for all enrolled patients.

**Results:**

Airway bleeding was reported in eleven patients of the positive group (11/29), with seven displaying bloody sputum or hemoptysis, and four featuring massive sputum crust formation in the trachea that resulted in dyspnea, and only one patient in the negative group (1/15), with a significant difference in the airway bleeding rate (37.9% vs. 6.7%, *p* < 0.05). The *SARS-CoV-2* RNA test results showed a statistical difference in cycle threshold (Ct) values between oropharyngeal swabs and tracheal swabs (*p* < 0.05).

**Conclusions:**

After tracheostomies, patients are more susceptible to airway bleeding if they are infected with *SARS-CoV-2*. The findings signify that in addition to droplet transmission through tracheostoma, *SARS-CoV-2* may infect the oropharynx by airborne and close contact transmission, and that given the higher viral load and longer infection time in the trachea, tracheal swabs are more reliable for *SARS-CoV-2* detection in these patients.

## Background

The Corona Virus Disease 2019 (COVID-19) pandemic caused by Severe Acute Respiratory Syndrome Coronavirus 2 (*SARS-CoV-2*) has devastated the global public health since December 2019 [1]. *SARS-CoV-2* is a coronavirus belonging to the genus *β*. It is a single-stranded RNA virus that encodes structural proteins including the spike (S) protein, envelope (E) protein, membrane (M) protein and nucleocapsid (N) protein [2, 3]. Since November 2021, the *Omicron* variant has spread rapidly worldwide as a result of its exceptional transmissibility, infectivity, and immune evasion [4]. In clinical practice, airway bleeding is observed in patients after temporary or permanent tracheostomy if they are infected with *SARS-CoV-2*. Anatomically, the upper and lower airways are separated after tracheostomy, depriving the patients of their physiological nasal functions. Does the direct passage of air through the tracheostoma increase the risk of respiratory diseases such as airway bleeding after a *SARS-CoV-2* infection?

The aftermath of *SARS-CoV-2* infection may be manifested variably, including the rare but grave hemorrhagic incidents [5]. A multicenter retrospective study has documented an overall bleeding rate of 4.8% in patients infected with *SARS-CoV-2*, including gastrointestinal bleeding, hemoptysis, oral mucosa bleeding, epistaxis, intracranial hemorrhage, pulmonary hemorrhage, etc., with the critically ill patients reporting a higher incidence of bleeding [6]. However, little has been available regarding airway bleeding events in patients after temporary or permanent tracheostomy if they are infected with *SARS-CoV-2*.

After tracheostomies, the anatomical changes in the airway and different respiratory specimens can impact the RNA test result if the patients are infected with *SARS-CoV-2*. A retrospective study has reported consistent *SARS-CoV-2* RNA test results for nasopharyngeal swabs and tracheal swabs in two-thirds of forty-five patients after tracheostomy but inconsistent results in one-third of the patients [7]. Other studies have documented a positive COVID-19 diagnostic test result for a nasopharyngeal swab but a negative result for a tracheal swab in patients who have undergone total laryngectomy and permanent tracheostomy [8, 9]. Therefore, controversies remain with regards to the selection of reliable respiratory samples for the *SARS-CoV-2* RNA test in patients who have received tracheostomies.

In this retrospective study, we investigated the correlation between airway bleeding and *SARS-CoV-2* infection in patients after temporary or permanent tracheostomy. We further evaluated the consistency of *SARS-CoV-2* RNA test results of the upper and lower airway samples in this population to justify a reliable sample selection. The findings may provide some novel insights into the management of airway hemorrhagic events in the context an infectious epidemic.

## Methods

### Patients and data collection

This retrospective study recruited forty-four patients who visited the First Affiliated Hospital of Fujian Medical University and underwent temporary or permanent tracheostomy between December 2022 and February 2023. Inclusion criteria were as follows: (1) reception of temporary or permanent tracheostomy for more than 7 days; (2) oropharyngeal swabs on admission; (3) at least 18 years of age. Exclusion criteria were as follows: (1) a history of chronic pulmonary disease; (2) a history of chronic heart failure; (3) a history of hemopathy resulting in spontaneous bleeding, such as leukemia, lymphoma, hemophilia, etc., and coagulation abnormalities; (4) tumors of the trachea, bronchi, and lung. Airway bleeding events were recorded for all enrolled patients. Demographic and clinical data were collected, including age, gender, endoscopic images of the trachea, computed tomography of the lung, pneumonia, hypertension, diabetes, chronic kidney failure, malignancy, and history of anticoagulant use. The study protocol was reviewed and approved by The Ethics Committee of First Affiliated Hospital of Fujian Medical University (Certificate NO.: [2015]084 − 2).

### *SARS-CoV-2* RNA detection

*SARS-CoV-2* RNA from respiratory samples was detected by Reverse Transcription-Polymerase Chain Reaction (RT-PCR). The viral load was measured by RT-PCR cycle threshold (Ct) values of the viral N and ORF1ab genes, which are inversely related to the viral load [10, 11]. All *SARS-CoV-2* RNA tests were performed in the laboratory of the First Affiliated Hospital of Fujian Medical University with a Line Gene 9600 fluorescent quantitative PCR instrument (FDQ-96 A, Bioer Technology, China). According to the results of the RT-PCR Ct values of oropharyngeal swabs, subjects were divided into a positive group (Ct value < 35) and a negative group (Ct value ≥ 35). The oropharyngeal and tracheal swabs of the positive group were simultaneously re-collected for the detection of *SARS-CoV-2* RNA.

### Statistical analysis

Statistical analyses were performed with SPSS 22.0 (IBM). Continuous variables were compared by Student’s t-test or Mann-Whitney U test. Categorical variables were compared by the Chi-square test or Fisher’s exact test. *P* values less than 0.05 were considered statistically significant. Graphs were depicted with GraphPad Prism 8.0 software and Adobe Illustrator CS6 software.

## Results

### No statistical difference in demographic characteristics and pneumonia is evident between the positive and negative groups

Of the 44 recruited patients, 29 patients were enrolled in the positive group, of whom 21 patients received a temporary tracheostomy and 8 received total laryngectomy and permanent tracheostomy before enrollment. The negative group included fifteen patients, of whom 12 patients underwent temporary tracheostomy and 3 patients had permanent tracheostomy before enrollment. No statistical difference was found between the positive and negative groups in terms of gender, age, history of hypertension, diabetes, chronic kidney disease, and malignancy. Pneumonia was reported in 22 patients of the positive group and 7 of the negative group, with no significant difference in the incidence of pneumonia between the two groups (75.9% vs. 46.7%, *p* > 0.05) (Table 1).

**Table 1:** Demographic and clinical characteristics in patients receiving tracheostomies

Parameter	All (*n* = 44)	SARS-CoV-2 Positive (*n* = 29)	SARS-CoV-2 Negative (*n* = 15)	*p* Value
Age, years (mean ± SD)	63.1 ± 7.9	64.6 ± 8.2	60.4 ± 6.6	0.099
Male, n (%)	40 (90.9)	26 (89.7)	14 (93.3)	1.000
Temporary tracheostomy, n (%)	33 (75.0)	21 (72.4)	12 (80.0)	-
Permanent tracheostomy, n (%)	11 (25.0)	8 (27.6)	3 (20.0)	-
Airway bleeding, n (%)	12 (27.3)	11 (37.9)	1 (6.7)	**0.035**
Pneumonia, n (%)	29 (65.9)	22 (75.9)	7 (46.7)	0.053
Hypertension, n (%)	10 (22.7)	7 (24.1)	3 (20.0)	1.000
Diabetes, n (%)	10 (22.7)	8 (27.6)	2 (13.3)	0.452
Chronic kidney failure, n (%)	3 (6.8)	2 (6.9)	1 (6.7)	1.000
Malignancy, n (%)	36 (81.8)	23 (79.3)	13 (86.7)	0.695

### The positive group reports a higher incidence of airway bleeding than the negative group

**Fig. 1:**
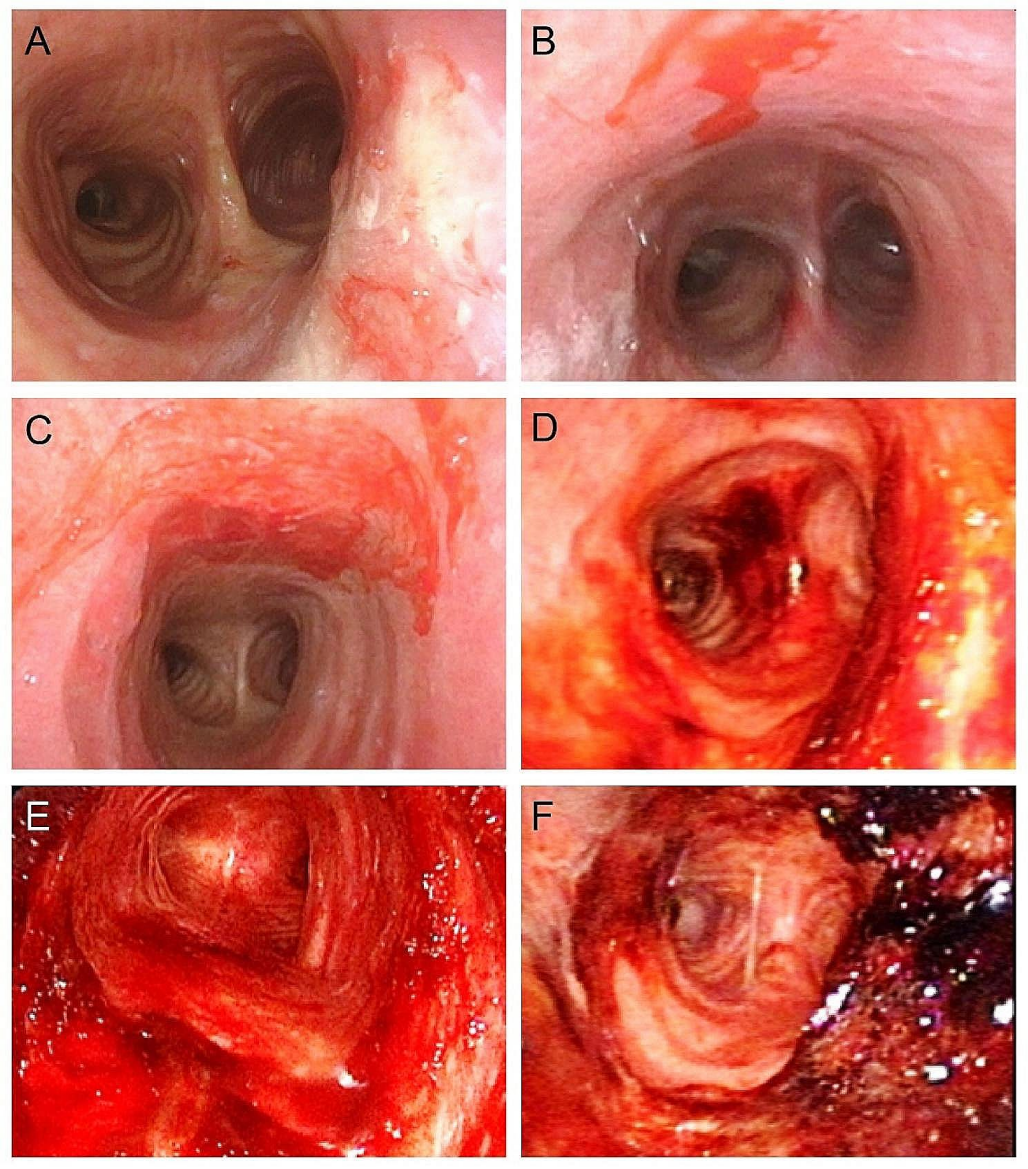
Airway bleeding events in patients receiving tracheostomies. **A**: Airway mucosal congestion and erosion. **B-C**: Airway mucosal erosion with minor bleeding. **D-E**: Diffuse bleeding of the airway mucosa. **F**: Crust formation in the airway

**Fig. 2:**
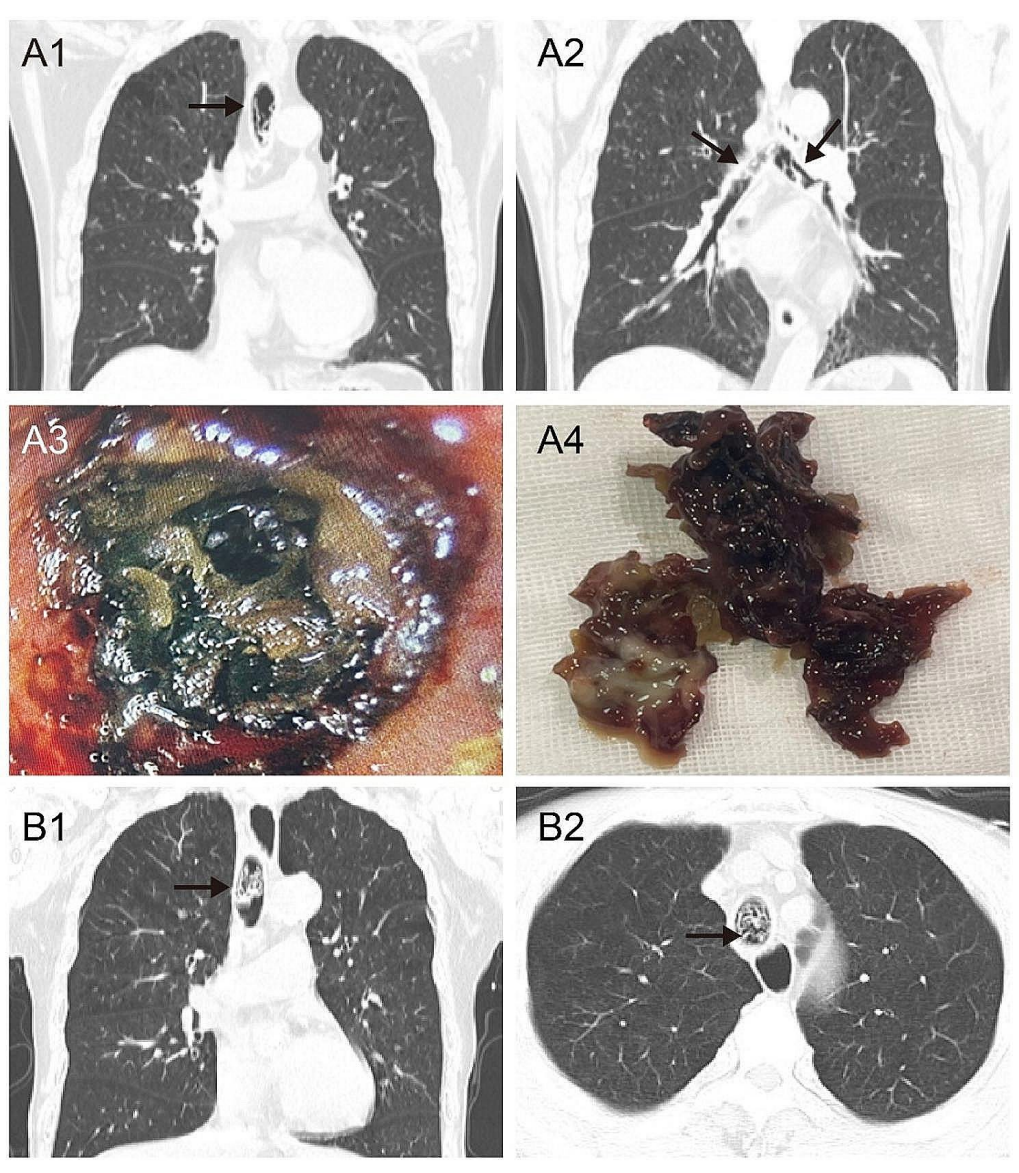
Sputum crust formation in patients receiving tracheostomies. **Case 1 (A1-4).** A 70-year-old male patient, 5 years after a total laryngectomy, was admitted with dyspnea for 2 days. The oropharyngeal and tracheal swabs were positive for *SARS-CoV-2.* After admission to the hospital, the patient underwent computed tomography (CT) scan of the lung and laryngoscope examination. **A1**: Dense shadow (indicated by black arrow) in the trachea as indicated by CT. **A2**: Dense shadow (indicated by black arrow) in the bronchus as indicated by CT. **A3**: Endoscopic manifestation of brown sputum crusts formation in the trachea causing airway obstruction. **A4**: Massive sputum crusts were removed from the airway by emergency surgery. **Case 2 (B1-2).** A 70-year-old male patient, 10 years after a total laryngectomy, was admitted with dyspnea for 3 days. **B1-2**: Dense shadow (indicated by black arrow) in the trachea as indicated by CT

**Fig. 3:**
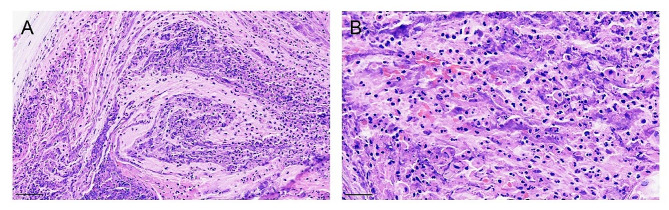
Hematoxylin-eosin staining of sputum crusts. **A**-**B**: The HE staining revealed a large amount of mucus, necrosis, inflammatory exudate containing neutrophils and eosinophils, and a small number of squamous epithelial cells in the sputum crusts. Scale bars, 60 μm (**A**), 30 μm (**B**)

In the positive group, airway bleeding was found in 11 patients (11/29), in which 7 cases reported bloody sputum or hemoptysis, with endoscopic manifestations of airway mucosa congestion, erosion, ruptured bleeding, and crust formation (Fig. 1), and 4 cases were admitted to the hospital for emergency removal of the endotracheal foreign body due to acute airway obstruction caused by massive sputum crust formation (Fig. 2). The sputum crusts were removed under the endoscope via the tracheostoma. The hematoxylin-eosin (HE) staining of sputum crusts revealed a large amount of mucus, necrosis, and inflammatory exudate containing neutrophils and eosinophils, and a small amount of squamous epithelial cells (Fig. 3). In the negative group, airway bleeding was found in only one patient (1/15). Further analyses indicated a statistical difference in the presence of airway bleeding between the positive and negative groups (37.9% vs. 6.7%, *p* < 0.05). In the positive group, 48.3% of patients had a history of prophylactic use of low molecular-weight heparin, with no significant difference in anticoagulant use between patients with and without airway bleeding (*p* > 0.05). The airway bleeding incidence was not statistically different between patients receiving temporary tracheostomy or permanent tracheostomy (*p* > 0.05) (Table 2).

**Table 2:** Demographic, clinical, laboratory characteristics in *SARS-CoV-2*-infected patients after tracheostomies

Parameter	Temporary tracheostomy (*n* = 21)	Permanent tracheostomy (*n* = 8)	*p* Value
Age, years (mean ± SD)	63.9 ± 7.6	66.1 ± 10.1	0.535
Male, n (%)	19 (90.5)	7 (87.5)	1
N-Ct values (mean ± SD)			
Oropharyngeal swab	29.81 ± 5.20	29.12 ± 4.73	0.523
Tracheal swab	24.45 ± 5.41	25.32 ± 5.06	0.838
ORF1ab-Ct values (mean ± SD)			
Oropharyngeal swab	30.63 ± 5.14	30.21 ± 4.26	0.377
Tracheal swab	25.48 ± 5.52	26.22 ± 5.29	0.99
Airway bleeding, n (%)	7 (33.3)	4 (50)	0.433
Pneumonia, n (%)	17 (81.0)	5 (62.5)	0.357
Anticoagulants, n (%)	11 (52.4)	3 (37.5)	-

N-Ct Values: cycle threshold values of *SARS-CoV-2* RNA obtained from N gene

ORF1ab-Ct values: cycle threshold values of *SARS-CoV-2* RNA obtained from ORF1ab gene

3.3 **The average Ct values of the tracheal swabs are lower than those of the oropharyngeal swabs.**

**Fig. 4:**
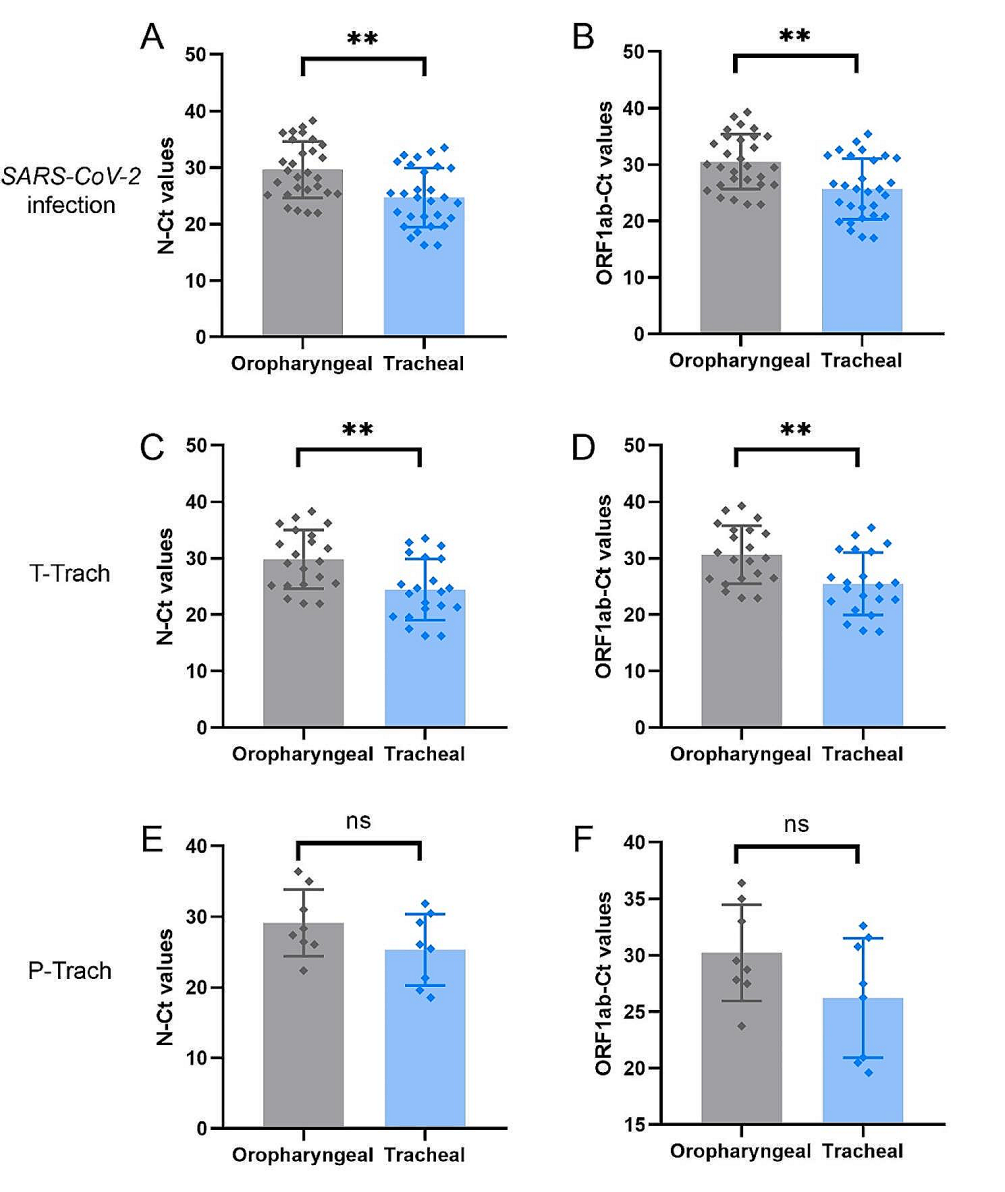
Detection of *SARS-CoV-2* RNA in oropharyngeal swabs and tracheal swabs from patients with *SARS-CoV-2* infection. **A-B**: swabs from patients with *SARS-CoV-2* infection (*n* = 29). **C-D**: swabs from patients receiving temporary tracheostomy (*n* = 21). **E-F**: swabs from patients receiving permanent tracheostomy (*n* = 8). T-Trach: temporary tracheostomy. P-Trach: permanent tracheostomy. N-Ct Values: cycle threshold values of *SARS-CoV-2* RNA obtained from N gene. ORF1ab-Ct values: cycle threshold values of *SARS-CoV-2* RNA obtained from ORF1ab gene. **P<0.05, ns: no significance. Statistical analysis was performed by Student’s t-test or Mann-Whitney U test

In the positive group, positive *SARS-CoV-2* RNA test results were reported in 22 patients for both oropharyngeal swabs and tracheal swab samples, and in 7 patients for tracheal swabs only. Further analyses of the oropharyngeal and tracheal swabs showed a significant difference in the average Ct values of the viral N gene (N-Ct values) (29.62 ± 4.99 vs. 24.69 ± 5.24, *p* < 0.05) and in the average Ct values of the viral ORF1ab gene (ORF1ab-Ct values) (30.51 ± 4.84 vs. 25.69 ± 5.37, *p* < 0.05) (Table 3; Fig. 4). However, no statistical difference in the Ct values of oropharyngeal swabs or tracheal swabs was found between patients after temporary tracheostomy and those after permanent tracheostomy (*p* > 0.05) (Table 2).

**Table 3:** Detection of *SARS-CoV-2* RNA in oropharyngeal swab and tracheal swab from patients with *SARS-CoV-2* infection

Ct values	Oropharyngeal swab	Tracheal swab	*p* Value
N-Ct values (mean ± SD)	29.62 ± 4.99	24.69 ± 5.24	**0.001**
ORF1ab-Ct values (mean ± SD)	30.51 ± 4.84	25.69 ± 5.37	**0.001**

N-Ct Values: cycle threshold values of *SARS-CoV-2* RNA obtained from N gene

ORF1ab-Ct values: cycle threshold values of *SARS-CoV-2* RNA obtained from ORF1ab gene

## Discussion

The current study investigated the association between airway bleeding events and *SARS-CoV-2* infection in patients receiving tracheostomies. We found that although the upper and lower airways were anatomically separated after temporary or permanent tracheostomies, positive *SARS-CoV-2* RNA test results were still reported for oropharyngeal swabs. The finding indicates that in addition to respiratory droplet transmission, *SARS-CoV-2* may infect the oropharyngeal epithelium by airborne and close contact transmission, which is consistent with a previous study [12]. As these patients do not manifest clinical symptoms such as fever, nasal congestion, runny nose, and sore throat, it is advisable for patients receiving temporary or permanent tracheostomy to wear surgical masks to cover the mouth, nose, and tracheostoma. Furthermore, after tracheostomies, positive *SARS-CoV-2* RNA test results were reported not only for the oropharyngeal swabs but also for tracheal swab samples, with a lower Ct value in the tracheal swabs than in the oropharyngeal swabs. The tracheal swabs were still tested positive even when *SARS-CoV-2* RNA test results of the oropharyngeal swabs were negative. These results suggest that even after tracheostomies, the trachea still may have a higher viral load and longer infection time than the oropharynx, which is consistent with the previous study [13]. For patients who received total laryngectomy and permanent tracheostomy while infected with *SARS-CoV-2*, no statistical difference in Ct values was evident between oropharyngeal and tracheal swabs, which may be attributed to the small sample size. As *SARS-CoV-2* RNA detection in the trachea and oropharynx can be discordant and the trachea may have a higher viral load, tracheal swab can be a more reliable specimen for *SARS-CoV-2* detection in patients after temporary or permanent tracheostomy.

Previous studies have documented that *SARS-CoV-2* infection can induce a severe procoagulant state despite prophylactic anticoagulation, with many adults developing myocardial infarction, cerebral infarction, and venous thromboembolism (VTE) [14, 15]. Additionally, it can incur rare but serious hemorrhagic conditions, including gastrointestinal bleeding [16], spontaneous cerebral hemorrhage [5], and spontaneous bleeding from the nasal and oral mucosa [17]. However, so far, bleeding events in patients who were infected with *SARS-CoV-2* after tracheostomies are even more rarely reported. In this study, 11 of 29 *SARS-CoV-2* infected patients (37.9%) reported airway bleeding events after receiving tracheostomies, which included bloody sputum, hemoptysis, and massive endotracheal crust formation.

To date, the mechanism of airway bleeding is not fully elucidated and various explanations have been proposed, including the status of tracheostomies, the virus itself, the viral inflammation, etc. In patients receiving temporary or permanent tracheostomy, the nasal mucociliary clearance of pathogens and particles is compromised [18], which may increase the risk of *SARS-CoV-2* infection during the epidemic [19]. Meanwhile, the mucosal production decreases and the viscosity of secretion increases in the trachea, making it easier to form sputum crusts in the airway [8], which often triggers severe cough and aggravates airway bleeding. Besides, according to the genetic data from the Global Initiative of Sharing All Influenza Data (GISAID) international database (https://gisaid.org/phylodynamics/china-cn/), from December 2022 to February 2023, Omicron variants were the dominant strains in Fujian, China. Compared with *SASR-CoV-2* wild-type (WT) or other variants, the *Omicron* variant of *SARS-CoV-2* mainly infects cells in the upper airway, bronchi, and trachea by binding the spike (S) protein to the main receptor, angiotensin-converting enzyme 2 (ACE2), and entering host cells via the endosomal route with the aid of ACE2 and cathepsin L [4, 20]. The single-cell sequencing shows that cells co-expressing ACE2 and cathepsin L are more abundant in the pharynx, trachea, and trachea than in alveolar epithelium [4]. The in vitro experiment shows that compared with the WT and *Delta* variants, the *Omicron* variant of the *SARS-CoV-2* displays a higher replication competence in bronchial tissues and lower replication competence in lung parenchymal tissues at 37 °C [21]. The reduced capacity of *Omicron* has also been observed in a polarized human lung epithelial cell model [22]. *Omicron* infection can cause damage, necrosis, and exfoliation of epithelium cells, leading to airway bleeding. In our study, we found patients infected with *SARS-CoV-2* showed no obvious clinical symptoms of pneumonia and extremely mild lesions in the lungs by the computed tomography (CT), which is consistent with the characteristics of *Omicron* infection [20]. In addition, *SARS-CoV-2* infection inhibits the interferon signaling pathway but elevates chemokine expression. As a result, when the virus multiplies exponentially, a large number of inflammatory cells are recruited to the site of infection by chemokines, leading to a cytokine storm that stimulates a serious inflammatory response, causing organ damage and airway bleeding [23, 24]. Chemokine can also cause activation, injury, and dysfunction of vascular endothelial cells, leading to bleeding or thrombotic events [18]. In our study, the HE staining of sputum crusts reported a large amount of mucus, necrosis, inflammatory exudate containing neutrophils and eosinophils, and a small amount of squamous epithelial cells, indicating airway inflammation.

After *SARS-CoV-2* infection, patients receiving temporary or permanent tracheostomy are more susceptible to airway bleeding and sputum crust formation. Therefore, when addressing a *SARS-COV-2*-infected patient, head and neck surgeons should take heed of the potential airway bleeding and adopt proper airway management. For patients receiving tracheostomies, more careful airway management should be prescribed, such as humidification, timely suction, and anticoagulant suspension when a bleeding tendency is evident.

Some limitations remain in this study. First, this was a single-center study that had a limited sample size. It will be necessary to evaluate airway bleeding events in a larger population. Second, no record has been made of the duration from virus infection to the airway bleeding and that of airway bleeding, which may be related to the viral load. However, for patients receiving temporary or permanent tracheostomy, our study provides important data on airway bleeding after *SARS-CoV-2* infection, which is useful in airway management during the epidemic.

## Conclusions

After temporary or permanent tracheostomy, patients are more susceptible to airway bleeding and sputum crust formation if they are infected with *SARS-CoV-2*, for *SARS-CoV-2* may infect the oropharynx by airborne and close contact transmission, as well as droplet transmission via tracheostoma. Therefore, surgical masks should be recommended for patients receiving tracheostomies to cover the mouth, nose, and tracheostoma, and head and neck surgeons should pay due attention to airway management when treating *SARS-CoV-2*-infected patients. Besides, tracheal swabs can be a more reliable specimen for *SARS-CoV-2* detection in these patients due to the higher viral load and longer infection time in the trachea.

## Data Availability

No datasets were generated or analysed during the current study.

## Abbreviations

COVID-19: Corona Virus Disease 2019
*SARS-CoV-2*: Severe Acute Respiratory Syndrome Coronavirus 2
RT-PCR: Reverse Transcription-Polymerase Chain Reaction
Ct: Cycle threshold
VTE: Venous thromboembolism
GISAID: Global Initiative of Sharing All Influenza Data
WT: Wild-type
S: Spike
E: Envelope
M: Membrane
N: Nucleocapsid
ACE2: Angiotensin-converting enzyme 2
CT: Computed tomography
HE: Hematoxylin-eosin
